# Usefulness of Frameless Neuronavigation–Guided Stereotactic Biopsy for Brain Lesions Under Local Anesthesia: Surgical Outcomes and Feasibility for Molecular Diagnosis—Case Series

**DOI:** 10.1227/neuprac.0000000000000133

**Published:** 2025-04-03

**Authors:** Sho Osawa, Makoto Ohno, Yasuji Miyakita, Masamichi Takahashi, Shunsuke Yanagisawa, Mai Honda-Kitahara, Takuma Nakashima, Shohei Fujita, Takahiro Tsuchiya, Tetsufumi Sato, Hirokazu Sugino, Akihiko Yoshida, Koichi Ichimura, Hiromichi Suzuki, Yoshitaka Narita

**Affiliations:** * Department of Neurosurgery and Neuro-Oncology, National Cancer Center Hospital, Tokyo, Japan;; ‡ Department of Neuro-Oncological Surgery, The Cancer Institute Hospital, Japanese Foundation for Cancer Research, Tokyo, Japan;; § Department of Neurosurgery, Tokai University School of Medicine, Tokyo, Japan;; ‖ Division of Brain Tumor Translational Research, National Cancer Center Research Institute, Tokyo, Japan;; ¶ Department of Anesthesiology and Intensive Care Medicine, National Cancer Center Hospital, Tokyo, Japan;; # Department of Diagnostic Pathology, National Cancer Center Hospital, Tokyo, Japan;; ** Department of Pathology, Kyorin University Faculty of Medicine, Mitaka, Japan

**Keywords:** Stereotactic brain biopsy, Neuronavigation, Local anesthesia, Molecular analysis, Case series

## Abstract

**BACKGROUND AND OBJECTIVES:**

Frameless neuronavigation–guided stereotactic biopsy (SB) is a common surgical technique for diagnosing intracranial lesions. A frameless SB is generally performed under general anesthesia; however, no reports are available on the efficacy and safety of frameless SBs under local anesthesia (LA). This study reports the surgical outcomes, diagnostic yield, and feasibility of molecular analyses after performing a frameless SB under LA (SB-LA).

**METHODS:**

The study retrospectively included patients who underwent a frameless SB-LA at our institute between March 2015 and January 2024. The clinical characteristics, intraoperative findings, completion rate of surgical procedure, complications, diagnostic yield, and feasibility of molecular analysis were analyzed retrospectively.

**RESULTS:**

The study included 80 patients. Surgical procedures were completed in 79 patients (98.7%); the diagnosis was confirmed in 76 cases (95.0%). The diagnoses included diffuse gliomas (n = 38, 47.5%), primary central nervous system lymphomas (PCNSL; n = 27, 33.8%), other brain tumors (n = 4, 5.0%), demyelinating diseases (n = 2, 2.5%), and normal brain/gliosis (n = 5, 6.3%). All samples were sufficient for basic molecular analyses of isocitrate dehydrogenase *1/2*, telomerase reverse transcriptase promoter, H3 histone family 3A, serine/threonine kinase B-RAF, and O-6-methylguanine deoxyribonucleic acid methyltransferase promoter methylation in gliomas and myeloid differentiation primary response gene 88 for PCNSLs. A comprehensive genomic profiling test using next-generation sequencing was attempted in 9 cases and was feasible in 8. Asymptomatic hemorrhages occurred in 14 patients (17.5%); no symptomatic hemorrhage occurred. Neurological deficits were observed in 1 patient (1.3%) who developed symptomatic small cerebral infarction. The median period from the first visit to our hospital to surgery was 3 days (range 0-12) for PCNSL and 6.5 days (range 0-21) for primary glioblastoma, isocitrate dehydrogenase wild-type.

**CONCLUSION:**

Frameless SB-LAs can be performed safely with a high diagnostic yield and feasibility for molecular analysis. Frameless SB-LAs improve early diagnoses and therapeutic interventions without compromising molecular information.

**ABBREVIATIONS:**
5-ALA: 5-aminolevulinic acid
*BRAF*
: serine/threonine kinase B-RAF
CGPT: comprehensive genomic profiling test
GA: general anesthesia
*H3-3A*
: H3 histone family 3A
*IDH*
: isocitrate dehydrogenase
LA: local anesthesia
*MYD88*
: myeloid differentiation primary response gene 88
NGS: next-generation sequencing
NOP: OncoGuide NCC OncoPanel System
NOS: not otherwise specified.
PCNSL: primary central nervous system lymphoma
SB: stereotactic biopsy
*TERT*
: telomerase reverse transcriptase.

Stereotactic biopsy (SB) is a useful surgical technique to establish diagnosis of intracranial lesions, especially in patients with multiple and/or deep lesions that are not amenable to resection.^1,2^ Frame-based SBs require image acquisition just before surgery.^3^ Frame-based SBs are safe and useful but time-consuming because of image acquisition after frame attachment. Frameless SBs preload preoperative images into a navigation system and determine spatial information by registering anatomic landmarks. Frameless SBs have a significantly shorter procedure time than frame-based SBs, with a comparable diagnostic yield.^4,5^ Robot-assisted SBs guide the biopsy needle to the target lesion according to a trajectory planned on preoperative images; their diagnostic yield and complication rate are comparable with those of frame-based and frameless SBs.^6,7^ However, considering the high initial cost of introducing robotic surgery, frameless SBs still remain the first choice for biopsy in many institutes.

Frame-based SBs are performed under general anesthesia (GA) or local anesthesia (LA), with no significant differences in diagnostic yield or safety.^8,9^ However, frameless SBs are commonly performed under GA.^4-6^ Therefore, it remains unclear whether frameless SBs under LA (SB-LA) can be performed safely with favorable diagnostic quality. To determine its clinical utility, this study retrospectively investigated the surgical and diagnostic outcomes of patients who underwent frameless SB-LAs.

## METHODS

### Participants

This retrospective case series included consecutive patients who underwent frameless SB-LAs at National Cancer Center Hospital between April 2015 and January 2024. The indication for frameless SB-LA was age ≥18 years, the SB could be performed in a supine or supine-lateral position, and the airway and respiratory status were stable and did not require oxygen administration. GA was indicated for brainstem lesions, except in 1 midbrain lesion case. We retrospectively analyzed age, sex, location, pathological diagnosis, target lesion size, the period between the first visit to our hospital and surgery, operative time, trajectory number, biopsy specimen number, completion rate of surgical procedure, diagnostic yield, and perioperative complications. Computed tomography was performed within 24 hours after surgery to evaluate postoperative hemorrhage, defined as any blood collection >5 mm in the minimal diameter within the biopsy trajectory.

Continuous variables are presented as medians and ranges. All participants provided written informed consent for inclusion before they participated in this study. This study was conducted in accordance with the Declaration of Helsinki, and the protocol was approved by our hospital's Research Ethics Review Committee.

### Local Anesthesia

Under noninvasive blood pressure monitoring, electrocardiography, and percutaneous oxygen saturation, 15 mg pentazocine and 5 mg diazepam were administered intravenously; additional doses were administered according to the sedation status. A 1% lidocaine solution was administered around the pin. The heads were fixed with a 3-pin Mayfield clamp with or without a horseshoe headrest. Horseshoe headrest was used to reduce fixation pressure and alleviate pain. Sedation and analgesia were initially managed by the neurosurgeon. We consulted an anesthesiologist in cases where the surgical procedure could not be continued because of restlessness or unstable vital signs (eg, systolic blood pressure >160 or <80 mm Hg, heart rate >130 or <60 bpm, or SpO_2_ <90% under 5 L/min oxygen administration).

### Surgical Procedure

Patients received 20 mg/kg 5-aminolevulinic acid (5-ALA) 3 to 6 hours before surgery.^10,11^ Preoperative T1 gadolinium contrast MRI or fluid-attenuated inversion recovery imaging, with or without contrast-enhanced computed tomography, was imported into StealthStation S7 or S8 (Medtronic Inc.). Registration was performed by tracing anatomic landmarks. Entry and target points were determined using Stealth neuronavigation software. Straight skin incisions and single burr holes were made at the entry points. A stealth biopsy needle was inserted stereotactically using a Vertek biopsy solution (Medtronic Inc.) under the guidance of a neuronavigation system. Biopsy specimens were obtained at the biopsy needle insertion position and positions rotated by 180°, 90°, or 270°. All biopsy specimens were subjected to intraoperative frozen sections, permanent sections, and genetic analyses. The fluorescence of 5-ALA was measured under 400- to 410-nm blue light. The wounds were closed after confirming the intraoperative diagnosis.

### Molecular Diagnosis

All samples were analyzed for mutations in isocitrate dehydrogenase (*IDH*) 1/2, telomerase reverse transcriptase (*TERT*) promoter, 1p/19q codeletion, H3 histone family 3A (*H3-3A*), and serine/threonine kinase B-RAF (*BRAF*), promoter methylation of O-6-methylguanine deoxyribonucleic acid methyltransferase (*MGMT*) for glioma, and mutation of myeloid differentiation primary response gene 88 (*MYD*88) for primary central nervous system lymphoma (PCNSL), as previously described.^12-14^

### Comprehensive Genomic Profiling Test

Patients with glioma potentially eligible to participate in the clinical trial underwent a comprehensive genomic profiling test (CGPT) using next-generation sequencing (NGS) panels after June 2019.^15^ CGPT was performed using the OncoGuide NCC OncoPanel System (National Cancer Center and Sysmex Corporation) or FoundationOne CDx Cancer Genomic Profile (F-One).

This case series has been reported in line with the PROCESS Guideline.^16^

## RESULTS

During the study period, 104 frameless SB procedures were performed. We excluded 24 patients who underwent frameless SB-GAs. The study included 80 patients who underwent frameless SB-LAs. The frameless SB-GA lesions included the brainstem (n = 9), cerebellum (n = 4), and 11 supratentorial lesions in the thalamus (n = 4), parietal lobe (n = 3), frontal lobe (n = 1), corpus callosum (n = 1), basal ganglia (n = 1), and temporal lobe (n = 1). We chose SB-GAs if the lesion was small (eg, in the thalamus) and the patient would not be able to tolerate surgery under LA because of stress.

The clinical characteristics of patients receiving SB-LAs are shown in Table 1. The median age was 65 years (range 19-88); 37 (46.3%) participants were male. Primary and recurrent disease occurred in 69 (86.3%) and 11 (13.8%) patients, respectively. The median diameter of the target lesions was 37 mm (range 11-100). Locations were frontal (n = 28, 35.0%), temporal (n = 12, 15.0%), parietal (n = 15, 18.8%), occipital (n = 1, 1.3%), insular (n = 1, 1.3%), multilobular (n = 4, 5.0%), basal ganglia (n = 5, 6.3%), thalamus (n = 7, 8.8%), corpus callosum (n = 5, 6.3%), cerebellum (n = 1, 1.3%), and brainstem (n = 1, 1.3%). The pathological diagnoses were glioblastoma, IDH wild-type (n = 22, 27.5%), astrocytoma, IDH-mutant (n = 3, 3.8%), oligodendroglioma, IDH-mutant and 1p/19q codeleted (n = 1, 1.3%), diffuse midline glioma, H3K27-altered (n = 1, 1.3%), glioma not otherwise specified (NOS) (n = 11, 13.8%), primary central nervous system lymphoma (PCNSL; n = 27, 33.8%), germinoma (n = 1, 1.3%), myeloid sarcoma (n = 1, 1.3%), ganglioglioma (n = 1, 1.3%), subependymoma (n = 1, 1.3%), demyelinating lesion (n = 2, 2.5%), normal brain tissue/gliosis (n = 5, 6.3%), and undiagnosed (n = 4, 5.0%). Postoperative MRI confirmed the target lesions in patients diagnosed with normal brain tissue or gliosis. Follow-up MRI showed no increase in the size of these lesions at a median follow-up of 4 months (range 0-31).

**TABLE 1.** Clinical Characteristics

Clinical characteristics	No. of patients (%)
Age, median, (range), y	65 (19-88)
Sex	
Male	37 (46.3)
Female	43 (53.8)
Primary	69 (86.3)
Recurrence	11 (13.8)
Histology	
Glioblastoma, IDH wild-type	22 (27.5)
Astrocytoma, IDH-mutant	3 (3.8)
Oligodendroglioma, IDH-mutant, and 1p/19q codeleted	1 (1.3)
Diffuse midline glioma, H3K27-altered	1 (1.3)
Glioma, NOS	11 (13.8)
Primary central nervous system lymphoma	27 (33.8)
Germinoma	1 (1.3)
Myeloid sarcoma	1 (1.3)
Ganglioglioma	1 (1.3)
Subependymoma	1 (1.3)
Demyelinating lesion	2 (2.5)
Normal brain tissue/gliosis	5 (6.3)
Undiagnosed	4 (5.0)
Location of the target lesion	
Frontal	28 (35.0)
Temporal	12 (15.0)
Parietal	15 (18.8)
Occipital	1 (1.3)
Insular	1 (1.3)
Multilobular	4 (5.0)
Basal ganglia	5 (6.3)
Thalamus	7 (8.8)
Corpus callosum	5 (6.3)
Cerebellum	1 (1.3)
Brainstem	1 (1.3)
Diameter of the target lesion, median, (range), mm	37 (11-100)

IDH, isocitrate dehydrogenase; NOS, not otherwise specified.

Repeat SB or tumor removal was performed in 4 cases 13 to 385 days after the first SB. In 3 of the 4 cases, strong contrast enhancement was observed, strongly suggesting a malignant brain tumor, and prompt reoperation was performed. The remaining case was initially monitored, but as the lesion gradually increased in size, reoperation was performed. The pathological diagnoses of these cases were glioblastoma, IDH wild-type (n = 2), medulloblastoma (n = 1), and glioneuronal tumor NOS (n = 1). All samples were sufficient for basic molecular analyses, including *IDH1/2*, *TERT* promoter, 1p/19q codeletion, *H3-3A*, *BRAF*, and promoter methylation of *MGMT*. In 38 patients with glioma, mutations in *IDH1/2* (n = 4, 10.5%), *TERT* promoter (n = 15, 39.5%), 1p/19q codeletion (n = 1, 2.6%), and *H3-3A* (n = 1, 2.6%) were detected. No mutation was detected in *BRAF*. Greater *MGMT* promoter methylation was detected in 17 (44.7%) cases. Mutation in *MYD88* was analyzed in 21 of 27 PCNSL cases and was detected in the 16 (76.2%) regions. Eleven glioma cases were classified as glioma NOS because genetic analysis showed no *IDH1/2*, *TERT* promoter, or *H3-3A* mutations. This study did not evaluate epidermal growth factor receptor amplification, gain of chromosome 7, and entire loss of chromosome 10. The pathological diagnoses of glioma NOS were high-grade astrocytoma (n = 10) and low-grade glioma (n = 1). CGPTs were attempted in 9 gliomas; 8 (88.9%) were feasible. OncoGuide NCC OncoPanel System and F-One were performed on 6 and 2 patients, respectively.

The median period from the first visit to our hospital to surgery was 3 days (range 0-12) for PCNSL and 6.5 days (range 0-21) for glioblastoma. Intraoperative findings are shown in Table 2. The median operative time was 79 minutes (range 37-167), including 30-45 minutes for intraoperative frozen tissue diagnosis. Three patients (3.8%) required anesthesiologist intervention: 2 showed restlessness, and 1 had elevated blood pressure. The surgery was terminated in 1 restless patient who underwent an SB-GA 13 days after the first SB; SB-LA was completed successfully in the other 2 cases. The median numbers of trajectories and biopsy specimens were 1 (range 1-4) and 4 (range 1-8), respectively.

**TABLE 2.** Operative Findings

Operative findings	No. of patients (%)
Duration of the surgical procedure, median, (range), min	79 (37-167)
Intervention by anesthesiologists	3 (3.8)
Discontinuation of surgery	1 (1.3)
No. of trajectories, median (range)	1 (1-4)
No. of biopsy samples, median (range)	4 (1-8)

To immediately confirm whether the tumor was successfully obtained, 5-ALA was administered preoperatively in cases suspected of malignant brain tumors with contrast-enhanced lesions. In 44 of 46 cases with contrast-enhanced lesions, the results of 5-ALA could be confirmed from the surgical records. Forty-three (97.7%) were positive for 5-ALA. In 1 case negative for 5-ALA, the pathological diagnosis could not be confirmed, and medulloblastoma was diagnosed using a sample obtained by a second surgery.

Complications are shown in Table 3. The morbidity and mortality rates were 1.3% and 0%, respectively. Asymptomatic hemorrhage occurred in 14 patients (17.5%); no symptomatic hemorrhage occurred. The median hematoma diameter was 8 mm (range 6-27). One patient (1.3%) developed a cerebral infarction postoperatively and presented with permanent hemiparesis. The case was a PCNSL that originated in the thalamus. After biopsy, the patient developed a cerebral infarction in the posterior limb of the internal capsule. It was considered to have been caused by vasospasm or dissection of the penetrating arteries, resulting from the negative pressure generated by the suction of tumor tissue. No infection or seizures were observed at the surgical site. Postoperative pneumonia and acute-phase deep venous thrombosis developed and recovered completely in 1 (1.3%) and 3 (3.8%) patients, respectively. No urinary tract infections occurred.

**TABLE 3.** Complications of Surgery

Complications	No. of patients (%)
Asymptomatic hematoma >5 mm in diameter	14 (17.5)
Diameter of the hematoma, median, (range), mm	8 (6-27)
Symptomatic hematoma	0 (0.0)
Cerebral infarction	1 (1.3)
Neurological deficit	1 (1.3)
Surgical site infection	0 (0.0)
Postoperative epileptic seizure	0 (0.0)
Postoperative pneumonia	1 (1.3)
Postoperative urinary tract infection	0 (0.0)
Postoperative deep venous thrombosis	3 (3.8)
Mortality	0 (0.0)

## DISCUSSION

This study is the first to investigate the outcomes of frameless SB-LAs in a large cohort. The diagnostic yield and morbidity rates were 95% and 1.3%, respectively. All the samples were sufficient for basic molecular analyses. CGPTs were feasible in 88.9% of cases. The favorable diagnostic yields, surgical outcomes, and feasibility of molecular diagnosis demonstrate the utility of frameless SB-LAs. Furthermore, by performing SB-LA, the median period from the first hospital visit to surgery was 3 days for PCNSL and 6.5 days for glioblastoma, enabling early treatment intervention.

Pathological analysis is necessary for the accurate diagnosis and treatment of intracranial lesions. Since the World Health Organization classification of central nervous system tumors incorporated molecular diagnostics into its diagnostic criteria in 2016, the need for tissue collection and molecular analysis has increased.^17^ We routinely performed *IDH1/2*, *TERT* promoter, and *H3-3A* analyses to diagnose diffuse gliomas that constitute most gliomas. In this series, 11 patients carrying none of the mutations in 4 genes mentioned above were diagnosed as glioma, NOS. To make a diagnosis based on the World Health Organization 2021 criteria, analytical methods targeting a few genes are limited, including Sanger sequencing, quantitative polymerase chain reaction, or multiplex ligation-dependent probe amplification; therefore, comprehensive genomic analysis using NGS is desirable.

CGPTs using NGS have been available in Japan under public health insurance coverage since June 2019; 18.5% of patients with gliomas who complete standard treatments can access at least 1 therapeutic agent by performing a CGPT.^15^ Although genetic analyses can be performed on glioma specimens obtained using an SB,^18-21^ the eligibility for a CGPT has not yet been reported. In this study, we showed that basic genetic testing was possible in all cases and also CGPTs were possible in 8 of 9 cases. These results suggest that even small specimens obtained by SB are sufficient for molecular diagnoses, including CGPTs.

Because managing body movement and blood pressure are sometimes difficult with LA compared with GA, an increased risk of undersampling and hemorrhage is a concern. Although reported SB diagnostic yields are 89.1% to 98%,^4,5,7,9^ our diagnostic yield for frameless SB-LAs was 95%. The mortality, morbidity, and symptomatic hemorrhage rates of frame-based SBs are 0.7%-4%, 3%-13%, and 0.9%-8.6%, respectively.^22^ The permanent neurological deficit of frameless SB-GAs is 1.6% to 2.5%.^4-6^ Although asymptomatic hemorrhages ≥5 mm in diameter occurred in 17.5% of our patients, no symptomatic hemorrhage occurred. Neurological deficits occurred in only 1.3% of patients. Thus, the diagnostic yield and safety of frameless SB-LAs were comparable with those frame-based SBs and frameless SB-GAs.

To our knowledge, there has been no study on the discontinuation rates of SB-LA. We assumed that LA could not control pain completely in some participants, causing restlessness and elevated blood pressure. Stress scores tend to be greater for SB-LAs than for SB-GAs.^8^ Therefore, improving pain control and sedation techniques and shortening the operative time would help minimize the patient burden. The latest frameless SB equipped with a robotic arm takes a median of 12 min for tissue samples under GA.^23^ Therefore, advances in surgical devices and anesthetic techniques are required.

Early diagnosis and treatment interventions for rapidly growing tumors such as PCNSL and glioblastoma can prolong survival.^24,25^ Compared with surgery under GA, surgery under LA lowers the labor and time required for anesthesia induction, facilitating scheduling and earlier surgery. Early diagnosis within 2 weeks and surgical intervention within 3 weeks of symptom onset can prolong survival in glioblastoma.^24^ A treatment delay of >30 days strongly correlates with shorter survival in PCNSL.^25^ Our analysis indicates that frameless SB-LA allows early diagnoses and therapeutic interventions, possibly improving prognoses, especially for malignant brain tumors.

### Limitations

One limitation of this study is its retrospective, single-center, single-arm analysis. Although we believe that SB-LA facilitated scheduling and contributed to early treatment intervention for malignant brain tumors, we acknowledge that the situation may vary depending on the institution or region. If GA can be performed promptly, SB-GA may be a more suitable. In addition, we generally perform frameless SB-GAs for brainstem or cerebellar lesions in the prone position. Because even a small amount of hemorrhage or contusion may cause severe complications in brainstem lesions, we consider SB-GAs to be preferable for brainstem lesions.

## CONCLUSION

Frameless SB-LAs can be performed safely and accurately. Although improvements in local anesthetic techniques are necessary, frameless SB-LA is a useful surgical technique for early diagnosis and therapeutic intervention without compromising molecular information.
